# Etiology of Ischemic Strokes of Patients with Atrial Fibrillation and Therapy with Anticoagulants

**DOI:** 10.3390/jcm9092938

**Published:** 2020-09-11

**Authors:** Jan C. Purrucker, Kyra Hölscher, Jennifer Kollmer, Peter A. Ringleb

**Affiliations:** 1 Department of Neurology, Heidelberg University Hospital, 69120 Heidelberg, Germany; hoelscher@stud.uni-heidelberg.de (K.H.); Peter.Arthur.Ringleb@med.uni-heidelberg.de (P.A.R.); 2 Department of Neuroradiology, Heidelberg University Hospital, 69120 Heidelberg, Germany; jennifer.kollmer@med.uni-heidelberg.de

**Keywords:** anticoagulant drugs, cerebral stroke, vitamin K antagonist, antithrombins, factor Xa inhibitors

## Abstract

Background: Reducing the number of ischemic strokes in patients with atrial fibrillation despite oral anticoagulation remains an important, yet largely unsolved challenge. Therefore, we assessed the etiology of ischemic strokes despite anticoagulation with vitamin K antagonists (VKA) or non-VKA oral anticoagulants (NOACs). Methods: Patients with known atrial fibrillation (AF), treatment with VKA or NOAC, and acute ischemic stroke admitted between 2015 and 2018 (1st half) were identified from the hospital database. Brain imaging data were independently reviewed. An integrated etiologic classification according to the ASCOD system was made. Medication errors (admission INR <2.0 in the VKA- or NOAC-specific concentration <10 ng/mL) or dosage/dosing errors were also analyzed. Results: Of 3610 patients screened, *n* = 341 were included (VKA, *n* = 127; NOAC, *n* = 214). An overall increasing rate of OAC-associated stroke per year was observed. In 95.3% of patients with adequate diagnostic work-up (*n* = 321/337), at least one additional potential, uncertain, or unlikely non-cardiac cause of stroke was identified. More patients in the VKA than in the NOAC group had a medication error (81/127, 63.8% vs. 102/205, 49.8%; *p* = 0.013). Conclusions: Stroke risk factors despite atrial fibrillation were highly prevalent. Although less common with NOACs than VKAs, medication errors are still frequent.

## 1. Introduction

Oral anticoagulation effectively reduces the risk of ischemic stroke in patients suffering from non-valvular atrial fibrillation (AF) compared to placebo or platelet inhibition [1,2]. Interestingly, while non-vitamin K antagonist oral anticoagulants (NOAC) have been shown to reduce the relative risk of intracerebral hemorrhage compared to vitamin K antagonists (VKA) by 50%, the relative risk of ischemic stroke despite oral anticoagulation has remained largely unchanged [3]. Indeed, a significant proportion of patients experience ischemic stroke while receiving oral anticoagulation therapy; in the primary prevention subgroup analyses of the randomized-control-trials comparing NOACs with VKA in AF patients, 0.7% to 1.3% of the patients suffered from an ischemic stroke per year, while in the secondary prevention cohorts, the percentage of patients developing at least a second ischemic stroke/transient ischemic attack was 1.8% to 2.3% per year [4,5,6,7]. Notably, in the primary prevention analyses, ischemic strokes occurred 2.1 to 10.1 times more often than hemorrhagic strokes and 2.2 to 27.4 times more often in the secondary prevention cohorts [4,5,6,7]. Recently, a pooled analysis underlined that patients with AF and oral anticoagulation before stroke were at a higher risk of recurrent stroke than patients without anticoagulation [8]. Thus, reducing the number of recurrent ischemic strokes in patients with AF despite oral anticoagulation remains an important, yet largely unsolved challenge. Data deciphering the causes of ischemic strokes despite oral anticoagulation remain scarce, however.

Herein, we aim to provide a more profound understanding of the etiology of ischemic strokes despite oral anticoagulation in order to inform clinical practice as well as future interventional studies to address the unmet need of further reducing recurrent stroke events in patients with AF who receive oral anticoagulation.

## 2. Experimental Section

### 2.1. Patient Cohort Description, and Patient Involvement

Consecutive patients with acute ischemic stroke (ICD-10 I63.x) admitted to the certified Stroke Unit or NeuroIntensive Care Unit of the Heidelberg University Hospital between 2015 and 2018 (1st half) were screened. Inclusion criteria for further data analysis were age ≥ 18 years, known non-valvular AF (paroxysmal, persistent, or permanent), and therapy with oral anticoagulation (VKA or NOAC (i.e., dabigatran, rivaroxaban, apixaban or edoxaban)) at the time of stroke onset. Patients were excluded in case of early palliative care orders, known presence of additional, high cardio-embolic risks besides AF, or insufficient quality or availability of source data. Furthermore, patients with documented pause of OAC >3 days before the index stroke, recent start of OAC <3 days before stroke, or bridging-therapy (administration of low-molecular-weight heparin) were also excluded. The research questions were developed due to the daily demanding questions of patients regarding the reasons why they were suffering from stroke despite taking oral anticoagulation. Results will be disseminated within the patient council of our Department of Neurology, Heidelberg University Hospital. Approval was obtained from the ethics committee of the Medical Faculty of Heidelberg, Germany, before beginning this study (S-525/2018). Informed consent of individual participants was waived due to the observational retrospective design of this study according to local and EU data regulations.

### 2.2. Data Acquisition

Information about medical history, stroke severity and course, performance, and results of both diagnostic and imaging and treatment modalities were extracted from the digital hospital archive. All diagnostic and treatment decisions were made during routine care. Diagnostic evaluation and treatment were administered in accordance with international and national guidelines, as well as with local standard operating procedures (available in German at https://www.klinikum.uni-heidelberg.de/neurologische-klinik/neurologie-und-poliklinik/ueber-uns/downloads). Briefly, during emergency assessment, patients receive a brain CT or MRI; if large-vessel occlusion is suspected or recanalization therapy planned (i.e., intravenous thrombolysis and/or endovascular therapy), CT or MR angiography is conducted. CT/MRI perfusion is performed in selected cases only. During the 24- to ≥72-h acute stroke diagnostics and therapy, additional follow-up CT or MRI procedures, extra- and transcranial Doppler/duplex sonography, continuous ECG and blood pressure monitoring, and echocardiography (transthoracic or transesophageal) are initiated. Routine laboratory results of non-specific coagulation tests (activated partial thromboplastin time (aPTT), international normalized ratio (INR), and thrombin time) as well drug-specific tests (anti-factor Xa or hemoclot assay) and platelet counts obtained at admission are collected. As a cut-off to differentiate between lower than expected NOAC concentrations, the level is set at 30 ng/mL following considerations previously published in Drouet et al. [9], and we use a lower cut-off of 10 ng/mL for sensitivity analyses, both of which are applicable to all NOACs. Renal function is assessed according to creatinine levels and the estimated glomerular filtration rate is electronically calculated (eGFR) according to the Chronic Kidney Disease Epidemiology Collaboration (CKD-EPI) equation, or if not available, the Modification of Diet in Renal Disease (MDRD) equation. Routine laboratory testing also includes measurement of the glycated hemoglobin (HBa1c) and a lipid profile. If indicated, a CSF analysis is performed, as is vasculitis screening (anti-nuclear antibodies, anti-double-stranded DNA, anti-neutrophil cytoplasmic antibodies, cardiolipin antibodies, C3c and C4 complement, and lupus anticoagulants).

### 2.3. Imaging Analysis

Brain CT or 3T MRI examinations were performed as part of local routine testing. Images were reviewed by a board-certified neuroradiologist (JK) and independently cross-checked by a board-certified neurologist (JP). Readers were blinded to all patient characteristics except for admission date. Lacuna was defined as a subcortical infarct smaller than or equal to 1.5 cm (≤2.0 cm on MRI diffusion images) in the largest dimension and in the distribution of the small, penetrating cerebral arteries, and with a fluid attenuated inversion recovery sequence (FLAIR) rim hyperintensity and parenchymal void (in follow-up) [10]. Acute infarcts were considered potentially non-embolic only if a lacuna or a small singular subcortical infarct was observed and there was a sign of small-vessel disease. White-matter changes were evaluated according to the Fazekas score (from 0 (no hyperintensity) to 3 (irregular periventricular hyperintensity extending into the deep white matter or large confluent lesions)) [11]. There was a good interrater correlation (0.7, 95% CI 0.61–0.72). For the analyses, the arithmetic mean of each of the of the two raters’ assessment was used.

### 2.4. Etiologic Classification

By integrating all diagnostic results, including the independent image analysis and clinical syndrome, one etiology or competing etiologies of the current stroke were classified according to ASCOD phenotyping (A: atherosclerosis; S: small-vessel disease; C: cardiac pathology; O: other causes; and D: dissection) [12]. Within ASCOD, the likelihood that every potential disease is causal for the stroke is graded in a 3(+2)-step system (1, potentially causal; 2, uncertain causality; 3 unlikely causal link; 0, disease is absent; 9, insufficient work-up to grade the disease). As all patients included in the current study had AF, we set C = 1 *a priori*, but investigated to determine any further cardio-embolic sources. Owing to the fact that a clinical syndrome suggesting a deep branch artery stroke was allowed to be classified as an uncertain potential cause in small-vessel disease without evidence on CT or MRI of a recent infarct (ASCOD, S2), acute stroke symptoms were classified according to the Oxford Community Stroke Project Classification [13]. Pure motor stroke, pure sensory stroke, sensorimotor stroke, and ataxic hemiparesis were considered as a lacunar syndrome.

### 2.5. Data Analysis

The distribution of continuous data is described as mean (SD) or median (interquartile ranges); for categorical data, absolute and relative frequencies (count and percentage) are reported. The Kolmogorov–Smirnov test was used to ascertain distribution of data. To compare proportions of demographic and clinical characteristics between patients with VKA and NOAC, the Fisher exact test was used. To compare continuous variables, the t-test or the non-parametric Mann–Whitney test was used, according to the skewness of the data. Admission time stamps were grouped to four time-intervals (0–6, 6–12, 12–18, and 18–24 h) for comparing drug-specific concentration levels. The intraclass correlation co-efficient was calculated to evaluate the reliability of the two raters’ scorings (two-way mixed, single-measure, or absolute agreement). All statistical tests are descriptive and no adjustment for multiple comparisons was made. Tests were 2-sided, and a *p* < 0.05 is regarded as significant. Analyses were conducted using IBM SPSS Statistics, version 25 and 26 (IBM Corp., Armonk, NY, USA).

## 3. Results

**Figure 1:**
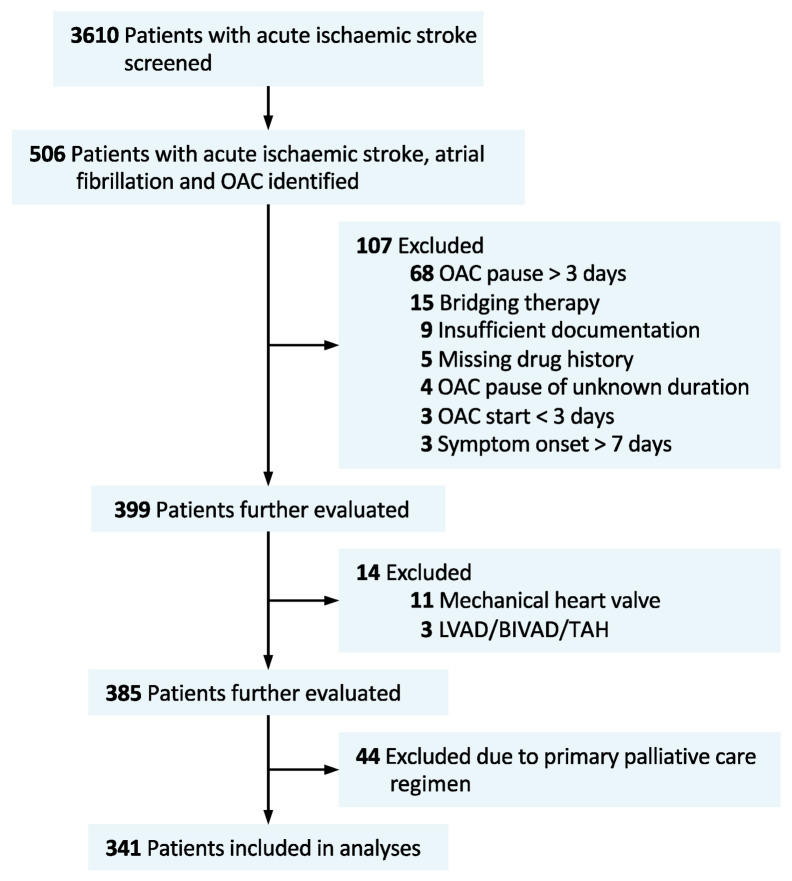
Flow chart of study population. OAC, oral anticoagulation; LVAD, left ventricular assist device; BIVAD, biventricular assist device; TAH, total artificial heart.

Of 3610 patients suffering from acute ischemic stroke and admitted during the 3.5-year study period, *n* = 506 patients with AF plus therapy with oral anticoagulation were identified. After excluding *n* = 107 patients, mainly because of known pause of oral anticoagulation >3 days, and further excluding *n* = 14 patients with additional, high cardio-embolic risks (mechanical heart valve or assist devices) and *n* = 44 patients who did not receive a full diagnostic work-up due to palliative care decisions, *n* = 341 patients were included in the analyses (VKA, *n* = 127 (37%), and NOAC, *n* = 214 (63%)) (see Figure 1 for patient in-/exclusion flow chart). Patients and case characteristics were well balanced between VKA- and NOAC-treated patients (see Table 1 for full patient details). Approximately half of the NOAC patients were treated with low-dose NOAC prescriptions (49.2%, Table 1). Concomitant antiplatelet therapy was prescribed more often in the NOAC than in the VKA group (10.3% vs. 2.4%, *p* = 0.009). While a total of 115 patients (33.7%) received endovascular therapy (EVT), with no difference between the VKA and NOAC group, fewer patients in the NOAC group were treated with intravenous thrombolysis (IVT, VKA 22.8% vs. NOAC, 7.5%, *p* < 0.001).

### 3.1. Time Trends

**Figure 2:**
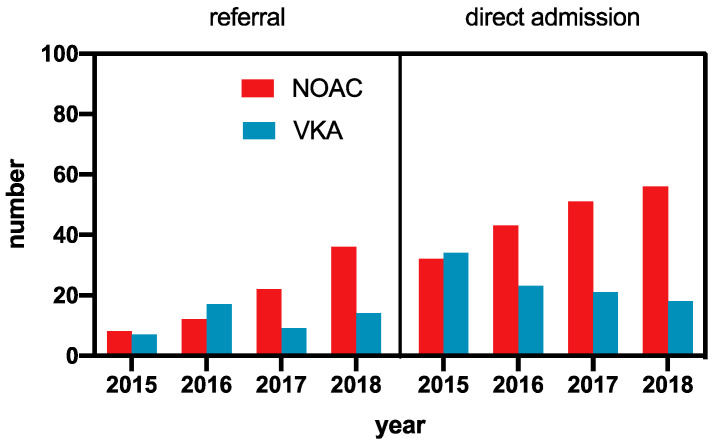
Admission time trend of patients with acute ischemic stroke and known AF and oral anticoagulation. VKA, vitamin K antagonist; NOAC, non-VKA oral anticoagulant. For 2018, data were extrapolated from the first half of the year.

While the absolute number of patients under VKA therapy suffering from an acute ischemic stroke decreased, a steeper increase in NOAC-treated patients was observed from 2015 to 2018, leading to an overall increase in OAC-associated ischemic strokes. The relative increase in NOAC-treated patients was independent of the admission type (external referral vs. direct admission), indicating a general trend (Figure 2).

### 3.2. Coagulation Status

A subtherapeutic INR <2.0 at admission was observed in 63.8% (*n* = 81) of the VKA-treated patients and an INR <1.8 in 52.8% (*n* = 67). INR values >3.0 were observed in 6.3% (*n* = 8). Those VKA patients with INR <2.0 had a median GFR of 75 mL/min (IQR 52–85), i.e., within the licensed GFR range for an alternative therapy with NOACs. For 88.3% (*n* = 189) of the patients with prescribed NOAC therapy, NOAC-specific quantitative coagulation testing was available at admission. NOAC concentrations ≤10 ng/mL were found in 22.9% (*n* = 49) and concentrations ≤30 ng/mL in 29.9% (*n* = 64) of the NOAC-treated patients. In a sensitivity analysis, performed due to the more rapid on- and offset of NOACs compared to VKA, we limited the analysis to patients admitted within a maximum of 12 h since “last-seen-well” (87.5% of *n* = 248 patients with clear documented “last-seen-well”). There was no difference in the fraction of patients with NOAC concentrations ≤10 ng/mL (*p* = 0.514), or ≤30 ng/mL (*p* > 0.99) respectively, between patients admitted within >12 h after “last-seen-well” and <12 h. In patients admitted in longer-time-windows, NOAC-specific tests were performed less frequently. Measured NOAC concentrations also did not differ between admission time intervals (*p* = 0.193). However, concentrations ≤10 ng/mL were determined in more patients receiving once-daily dosing than in patients with twice-daily dosing regimens (77.6% vs. 22.4%, *p* < 0.001).

### 3.3. Dosage Errors

In a total of 58 patients (31%) from the NOAC group for whom full documented medication schedules were available (*n* = 187, 87.4%), pre-stroke dosage errors were encountered. Of the 58 patients, *n* = 13 (22.4%) received a non-licensed dosage according to the most recent prescription labelling for prevention of stroke or systemic embolism in AF: OD instead of BD prescription, dabigatran *n* = 4, apixaban, *n* = 4; rivaroxaban was prescribed at 10 mg OD in *n* = 3 patients, and in one patient, 2.5 mg BD in combination with aspirin 100 mg per day was prescribed. In addition, one patient received a dose of 15 mg rivaroxaban BD (i.e., a higher than indicated dose) due to failure to reduce the dosage 21 days after a pulmonary embolism two months before stroke. Furthermore, in 45 patients, a heterogeneous pattern of further dosing errors was observed, mainly due to underdosing. Rivaroxaban-treated patients were more often underdosed (*n* = 23/86, 26.7%,) than patients receiving the factor Xa inhibitors apixaban (*n* = 9/59, 15.3%) and edoxaban (*n* = 1/14, 7.1%). A potential underdose was observed in *n* = 4 patients under dabigatran treatment (low dose despite age 75–80 years and GFR > 50 mL/min). In contrast, overdosing was observed less frequently among all patients (rivaroxaban, *n* = 5 and edoxaban, *n* = 2). Of patients without dosage errors and admission within 12 h of last-seen-well, 23.9% had NOAC levels ≤10 ng/mL at admission, indicating potential non-intake.

### 3.4. Medication Errors

The composite ‘medication error’ of either laboratory-based evidence of insufficient anticoagulation (here defined as INR < 2.0 in the VKA- or drug-specific concentration <10 ng/mL in the NOAC-group, respectively) and/or dosage or dosing errors was observed in 183/332 patients (55.1%), with more medication errors in the VKA (81/127, 63.8%) than in the NOAC group (102/205, 49.8%; *p* = 0.013).

### 3.5. Etiologic Classification of OAC-Associated Strokes

**Figure 3:**
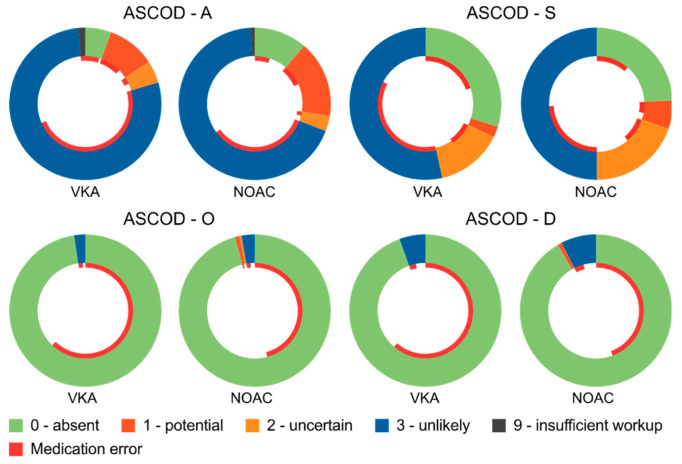
Visualization of the ASCOD categorization, including the relative fraction of patients with present ‘medication error’ with regard to each ASCOD category (inner red circle). Medication error was defined as either laboratory-based evidence of insufficient anticoagulation (here defined as INR < 2.0 in the VKA-, or drug-specific concentration < 10 ng/mL in the NOAC-group, respectively) and/or dosage or dosing errors. ASCOD, A: atherosclerosis; S: small-vessel disease; O: other causes; D: dissection. The category C (cardiac pathology) was set to 1 in all patients due to the known atrial fibrillation and, thus, is not presented. VKA, vitamin K antagonist; NOAC, non-VKA oral anticoagulant.

A cardiac source of embolism without any further non-cardiac cause of embolism or thrombosis (ASCOD: C =1, ASOD = 0) was found in 16 patients (4.7%; *n* = 4 VKA, *n* = 12 NOAC). In the remaining 95.3% of patients with an adequate diagnostic work-up (*n* = 321/337), there was at least one additional (a) potential, (b) uncertain, or (c) unlikely non-cardiac cause of stroke. Table 2 summarizes these causes. Briefly, a potentially atherosclerotic cause of stroke was considered (ASCOD: A = 1) in numerically fewer VKA- than NOAC-treated patients (VKA, 10.2% vs. NOAC, 16.5%, *p* = 0.147). Small-vessel disease was considered potentially causal (ASCOD: S = 1) in 16 patients (4.7%; *n* = 3 VKA, *n* = 13 NOAC, *p* = 0.148), and potentially causal or cause uncertain in 76 patients (22.3%) (Table 2). A rare, potentially causal disease (ASCOD: O = 1) was not found in any of the VKA- but was found in *n* = 2 of the NOAC-treated patients (polycythemia/thrombocythemia); neither of these two patients had been co-treated with antiplatelet agents at the time of stroke. An ipsilateral dissection was observed in *n* = 2 of the NOAC patients but in none of the VKA patients. Figure 3 provides a visualization of the ASCOD categorization, including the relative fraction of patients with present ‘medication error’ with regard to each ASCOD category. No incidence of a ‘medication error’ was found in 24/48 (50%) patients with a potentially atherosclerotic cause (ASCOD: A = 1) or in 8/16 patients (50%) with potentially causal small-vessel disease (ASCOD: S = 1).

## 4. Discussion

Our study investigated different etiologies of stroke in patients with known AF despite oral anticoagulation and reveals two major findings: (1) medication errors are observed in more than half of the patients, and (2) relevant non-cardiac causes of stroke are prevalent in up to one-fifth of the patients.

Low rates of anticoagulation due to practical concerns like, for example, the requirement of frequent routine INR monitoring to adjust the dosing schemes, were among the reasons triggering the development of NOACs. In patients treated with VKA, we observed INR below the therapeutic range (<2.0) in 64%. Since it has been previously shown that the relative benefit of NOACs over VKA, in terms of the reduction of vascular events, and mortality is increasing at centers with poor INR control compared to good control [14], a stringent INR monitoring, as well as early considerations to switch to NOACs if the INR is not achieving the therapeutic range, is recommended [15]. In contrast to VKA, routine monitoring is still not recommended in NOACs [9]. Indeed, according to our data, potential medication errors are reduced with NOACs compared to VKA in patients who suffer from stroke, despite oral anticoagulation, but continue to occur in approximately 50%.

Approximately one-fifth of the patients without formal dosage-error had even very low NOAC-levels (≤10 ng/mL), and actual non-intake of the prescribed medication must be assumed as the most probable reason for this finding. Further reasons for medication errors include a tendency to use lower than indicated doses, especially due to failures to adjust dosage according to current renal function. Rivaroxaban, the factor Xa inhibitor with the most variable available doses in different indications [16], was the NOAC showing the highest rate of medication errors. Another study recently reported a rate of up to 65% of patients with Factor Xa inhibitor treatment having received an inappropriate dose regimen before stroke [17]. Furthermore, lower plasma levels at admissions were associated with greater stroke severity [17,18]. Although different available doses principally enhance the possibility to better tailor individual medications, our data underline the growing need to educate prescribing physicians. Software-based systems might, by integrating diagnoses, weight, and GFR/creatinine levels, support physicians. 

We also observed that therapy with NOACs given on a once-daily dosing regimen led to a 3.5-fold relative increase in low (≤10 ng/mL) specific NOAC concentrations at admission compared to NOACs given twice daily. It is known that the peak-to-trough variability is larger for once-daily dosing; furthermore, single omitted doses lead to a greater decrease in plasma levels in a once-daily vs. a twice-daily regimen [19]. As it is extremely difficult in practice to manage patient adherence more closely, the broad implementation of smart tools reminding patients to take their drugs is important (reminder packaging) [20]. Data from larger population-based registries will help to gain more insight into the potential risks of once-daily regimens for NOACs with short half-lives, especially in patients with healthy kidneys.

Due to the observational nature of our study, we cannot prove that medication errors caused the ischemic stroke event in individual patients or determine the level to which a concurrent risk factor contributed to the stroke. However, in 50% of the patients with a potentially atherosclerotic cause or relevant small-vessel disease, no medication error was detected. Risk factors such as arterial hypertension, diabetes, and chronic heart failure (i.e., risk factors for both atherosclerotic and small-vessel disease) further increase the risk of stroke in patients with AF, and thus, were integrated in the commonly used CHA_2_DS2VAsc score [21]. Currently available data cannot determine whether adding a low-dose antiplatelet to a NOAC would be beneficial or not in preventing recurrent stroke in patients with significant atherosclerosis and AF [22,23], a therapeutic option that warrants examination in a randomized clinical trial.

To make full use of stroke prevention, lowering low-density lipoprotein (LDL) levels should also be kept in focus. Current LDL level targets were only reached in a minority in our study, with 77% of the patients having LDL cholesterol levels ≥70 mg/dl (≥1.8 mmol/L), which is the recommended upper threshold according to recent guidelines for patients at high risk [24,25].

As a strength of our study, NOAC plasma levels at administration were available for 88% of patients from routine clinical testing; therefore, reliance on medication history alone was not necessary to determine potential medication errors. Due to the different pharmacokinetics of NOACs compared to VKA, it is impossible to extrapolate the actual level at the time of stroke-onset from a single measured value later on. Acknowledging the rapid on- and offset of NOACs, we performed a sensitivity analysis by limiting the analysis to those patients who were admitted within 12 h of last-seen-well before, but could not find any difference in the fraction of patients with low NOAC levels compared to patients arriving in later time-windows. This reflects a previously shown high variability of NOAC concentrations, even when similar intervals since last intakes are compared [26].

While we classified all our study data based on the existence of a potential cardiac etiology due to AF irrespective of medication status (as suggested by the ASCOD classification), this was handled differently in another observational study on stroke patients receiving NOAC [27].

Our study also has some limitations. We did not co-register current use of lipid-lowering therapies, such as statins, which might have pleiotropic effects in addition to the mechanistic function of lowering LDL [28]. Due to the observational nature of our study, all data are hypothesis-generating, but might help to inform future interventional trials. We did not include a matched cohort of non-stroke patients because the comprehensive work-up at the level done in stroke patients to unveil even less common cardiovascular diseases is not performed routinely in other diseases. While we observed a continuous increase in ischemic strokes despite NOAC therapy and a stabilizing rate of VKA-associated strokes, a population-based analysis would be necessary to elucidate the potential underlying reasons for this trend. An increase in oral anticoagulation therapies, in general, with preferred primary use of NOACs, and consequently increasing stroke rates would correspond with results from registries [29].

## 5. Conclusions

Medication errors in patients with AF suffering from ischemic strokes despite therapy with oral anticoagulants are still common, despite the introduction of NOACs. Concurrent risk factors, such as atherosclerotic disease and hypercholesterinemia are not treated adequately. Interventional trials need to determine whether better patient education, e.g., in conjunction with smart devices to increase therapy adherence, as well as a more stringent follow-up of these patients with AF to optimize concurrent risk factor treatment would be sufficient to reduce the risk of stroke in these high-risk group.

## Figures and Tables

**Table 1:** Patients and case characteristics (*n* = 341).

	VKA (*n* = 127)	NOAC (*n* = 214)	*p*-Value
Age, mean (SD)	79.4 (8.0)	77.9 (8.9)	0.119
Women	57 (44.9)	102 (47.7)	0.654
NOAC			-
Apixaban	-	65 (30.4)	
Dabigatran	-	34 (15.9)	
Edoxaban	-	14 (6.5)	
Rivaroxaban	-	101 (47.2)	
Low-dose NOAC	-	97/197 (49.2)	
Phenprocoumon	125 (98.4)	-	
Concomitant antiplatelet therapy	3 (2.4)	22 (10.3)	0.009
Comorbidities			
Arterial hypertension	112 (88.2)	195 (91.1)	0.455
Diabetes mellitus	47 (37.0)	71 (33.2)	0.482
Hyperlipidemia	51 (40.2)	67 (31.3)	0.101
Ischemic heart disease	46 (36.2)	75 (35.0)	0.907
Myocardial infarction	19 (15.0)	30 (14.0)	0.873
Peripheral artery disease	16 (12.6)	22 (10.3)	0.594
Stroke/TIA	39 (30.7)	81 (37.9)	0.198
Bleeding	6 (4.7)	14 (6.5)	0.635
Smoking	14 (11.0)	34 (15.9)	0.26
Malignancy, active	4 (3.3)	12 (5.9)	0.428
Renal function at admission			
GFR; mean (SD)	66.5 (22.3)	68.2 (20.3)	0.471
GFR < 50 mL/min	26 (20.5)	43 (20.1)	>0.99
GFR < 30 mL/min	9 (7.1)	8 (3.7)	0.201
Lipid levels, serum, mg/dl mmol/L			
HDL cholesterol	41 (34–51) 1.1 (0.9–1.3)	44 (35–52) 1.1 (0.9–1.3)	0.409
LDL cholesterol	90 (68–112) 2.3 (1.8–2.9)	97 (71–118) 2.5 (1.8–3.1)	0.202
LDL cholesterol ≥ 70 mg/dl (≥1.8 mmol/L)	89/120 (74.2)	161/204 (78.9)	0.34
Triglycerides	104 (72–129)	97 (71–128)	0.355
Onset (last-seen-well in case exact onset is unknown) to admission *, hours	3.7 (2.0–8.4)	3.5 (1.5–7.0)	0.445
Functional status			
Pre-stroke mRS	2 (1–3)	2 (1–3)	0.943
mRS at admission	4 (3–4)	3 (2–4)	0.251
mRS at discharge	3 (2–4)	3 (2–4)	0.799
NIHSS at admission	7 (3–17)	3 (3–16)	0.758
Imaging modality			
CT	123 (96.9)	210 (98.1)	0.477
MRI	32 (25.2)	54 (25.2)	>0.99
Large-vessel occlusion	61 (48.0)	109 (50.9)	0.654
IVT	29 (22.8)	16 (7.5)	<0.001
EVT	41 (32.3)	74 (34.6)	0.723
CAS	0 (0)	4 (1.9)	0.301
CEA	1 (0.8)	7 (3.3)	0.266

Data are median (IQR) or *n* (%) if not indicated otherwise. * exact onset or last-seen-well documented in *n* = 100 (VKA), and *n* = 148 (NOAC) cases. GFR, glomerular filtration rate; HDL, high-density lipoprotein; LDL, low-density lipoprotein; mRS, modified Rankin scale; NIHSS, National Institute of Health Stroke Scale; CT, computed tomography; MRI, magnet resonance imaging; IVT, intravenous thrombolysis; EVT, endovascular therapy; CAS, carotid artery stenting; CEA, carotid endarterectomy; VKA, vitamin K antagonist; NOAC, non-VKA oral anticoagulant.

**Table 2:** Etiologic classification.

Disease (Causality)	VKA (*n* = 127)	NOAC (*n* = 214)	*p*
Cardiac pathology (potential)	127	214	-
Atherosclerosis (potential)	13/125 (10.4)	35/212 (16.5)	0.147
Atherosclerosis (potential or uncertain)	19/125 (15.2)	42/212 (19.8)	0.309
Small-vessel disease (potential)	3 (2.4)	13 (6.1)	0.184
Small-vessel disease (potential or uncertain)	21 (16.5)	55 (25.7)	0.059
Other causes (potential)	0 (0)	2 (0.9)	0.531
Dissection (potential)	0 (0)	2 (0.9)	0.531
Insufficient work-up (N9)	2 (1.6)	2 (0.9)	0.63

Data are *n* (%). ASCOD, A: atherosclerosis; S: small-vessel disease; O: other causes; D: dissection. The category C (cardiac pathology) was set to potential (1) in all patients due to the known atrial fibrillation. Presence of any further disease category (in any grade) was allowed in all categories but N9. N9 = insufficient work-up in ≥1 of the ASCOD categories. VKA, vitamin K antagonist; NOAC, non-VKA oral anticoagulant.
